# Assessment of avidity related to IgG subclasses in SARS-CoV-2 Brazilian infected patients

**DOI:** 10.1038/s41598-021-95045-z

**Published:** 2021-09-03

**Authors:** Andrew D. Moura, Hernan H. M. da Costa, Victor A. Correa, Ana K. de S. Lima, José A. L. Lindoso, Elizabeth De Gaspari, Marisa A. Hong, Jair P. Cunha-Junior, Carlos R. Prudencio

**Affiliations:** 1 Center of Immunology, Institute Adolfo Lutz, São Paulo, Brazil; 2 Institute of Infectology Emilio Ribas, São Paulo, Brazil; 3 Department of Infectious Disease, School of Medicine, São Paulo University, São Paulo, Brazil; 4 Laboratory of Protozoology, Institute of Tropical Medicine, São Paulo, Brazil; 5 Laboratory of Immunochemistry and Immunotechnology, Department of Immunology, Federal University of Uberlândia, Uberlândia, MG Brazil

**Keywords:** Immunological techniques, Diagnostic markers, Applied immunology, Infectious diseases

## Abstract

SARS-CoV-2 is considered a global emergency, resulting in an exacerbated crisis in the health public in the world. Although there are advances in vaccine development, it is still limited for many countries. On the other hand, an immunological response that mediates protective immunity or indicates that predict disease outcome in SARS-CoV-2 infection remains undefined. This work aimed to assess the antibody levels, avidity, and subclasses of IgG to RBD protein, in symptomatic patients with severe and mild forms of COVID-19 in Brazil using an adapted in-house RBD-IgG ELISA. The RBD IgG-ELISA showed 100% of specificity and 94.3% of sensibility on detecting antibodies in the sera of hospitalized patients. Patients who presented severe COVID-19 had higher anti-RBD IgG levels compared to patients with mild disease. Additionally, most patients analyzed displayed low antibody avidity, with 64.4% of the samples of patients who recovered from the disease and 84.6% of those who died in this avidity range. Our data also reveals an increase of IgG1 and IgG3 levels since the 8th day after symptoms onset, while IgG4 levels maintained less detectable during the study period. Surprisingly, patients who died during 8–14 and 15–21 days also showed higher anti-RBD IgG4 levels in comparison with the recovered (P < 0.05), suggesting that some life-threatening patients can elicit IgG4 to RBD antibody response in the first weeks of symptoms onset. Our findings constitute the effort to clarify IgG antibodies' kinetics, avidity, and subclasses against SARS-CoV-2 RBD in symptomatic patients with COVID-19 in Brazil, highlighting the importance of IgG antibody avidity in association with IgG4 detection as tool laboratory in the follow-up of hospitalized patients with more significant potential for life-threatening.

## Introduction

COVID-19 (*Coronavirus disease* 2019), the most recent pandemic caused by severe acute respiratory syndrome-related coronavirus 2 (SARS-CoV-2), resulting in an exacerbated crisis in the health public, declared as a global emergency by World Health Organization (WHO)^1^ in March 2020. SARS-CoV-2 was first reported in Wuhan, China, in December 2019, resulting in a large number of individuals presenting symptoms such as fever, dry cough, dyspnea, body pain, loss of taste and smell, and sometimes atypical pneumonia that might be fatal in a small percentage of cases (around 5%)^2–4^. Brazil became the epicenter of COVID-19 in June of 2020 and nowadays Brazil has experienced a rise in the number of cases and deaths associated with SARS-CoV-2 infection^5,6^. This scenario may impose new challenges to health services shortly, including the requirement for novel rapid diagnostic tools to interrupt the COVID-19 epidemiological chain^7,8^.

SARS-CoV-2 is a novel human-infecting betacoronavirus that is enveloped, non-segmented, positive-sense single-stranded RNA of around 65–125 nm diameter with crown-like spikes proteins on its outer surface^9^. The new coronavirus genome encodes nonstructural proteins from two open reading frames 1a and 1b (open reading frame 1—ORF1), and structural proteins, such as spike (S), small envelope (E), membrane (M), and nucleocapsid (N) glycoprotein^10^. The S glycoprotein is a transmembrane protein with a molecular weight of 150 kDa that binds to the angiotensin-converting enzyme 2 (ACE2) or other candidate receptors expressed on the host cells surface through receptor binding domain (RBD) presented in the S1 subunit of S protein^11,12^. Studies reveal that the higher infectivity capacity of SARS-CoV-2 compared to other coronavirus is due to genomic mutations on the RBD domain from the S protein that plays an essential role in virus attachment and invasion to the host cell^9,13–15^.

Real-time reverse transcription-polymerase chain reaction (RT-PCR) is a useful assay for the detection of viral genomes in biological samples, including pharyngeal and saliva specimens from COVID-19 patients. Although RT-PCR is a very sensitive diagnostic test for detect COVID-19 positive patients, its application is limited to a narrow time window defined by the presence of the virus in mucosal secretions^16–18^ and also by the need to manipulate infectious biological samples in biosafety level -2 (BSL-2) structure. Therefore, the development of new serological tests based on immunodominant SARS-CoV-2 antigens is an urgent need in the pandemic^19^. In this context, a wide set of serological tests have been applied mainly as an essential diagnostic tool to evaluate the positivity of SARS-CoV-2. Nevertheless, their accuracy is inferior to RT-PCR in the initial phase of SARS-CoV-2 infection^13,16,20–23^.

RBD domain from SARS-CoV-2 spike protein is highly immunogenic and induces IgG antibody response in acutely and convalescent infected patients, it is considered a potential target for serological assays^22,24–28^ and vaccine development^10,29–32^. Although there are advances in vaccine development^33–35^, it is still limited for many countries. On the other hand, the immunological response in SARS-CoV-2 infection remains undefined, with several data still emerging in the scientific literature concerning this new coronavirus pandemic^36–39^. While virus nucleic acid is detected between 3–10 days after infection, antibody production can only be detected few  days after symptoms onset, similar to SARS-CoV-1 infection^38^. A high level of antibodies is observed in patients with severe disease^40,41^. However, data regarding their ability to neutralize infection, as well as the presence of IgG subclasses (IgG1, IgG2, IgG3, and IgG4) is missing. In contrast, some recovered, or asymptomatic patients produce low or absence of IgG levels against nucleoprotein and spike protein from SARS-CoV-2^42^. Antibodies IgG1 and IgG3 are related to complement fixation, antibody-dependent cellular toxicity, and viral neutralization^41^, and a recent study has demonstrated that IgG1 and IgG3 specific to RBD occurs primarily in COVID-19 patients during acute infection. At the same time, IgG2 and IgG4 are less detectable in patients sera^41,43,44^, suggesting IgG subclasses as an important marker to distinguish disease time-point and, perhaps, disease severity.

Other factors can also contribute to the understanding of humoral responses to SARS-CoV-2 and the role of neutralizing antibodies in the convalescent plasma of donors, such as an association between antibody neutralizing titers and antibody avidity^45^. Antibody avidity is defined as the strength of bivalent or multivalent interaction between antibody and epitope^46^. It is well described that antibody avidity increase over duration of infection and remained elevated, as expected to SARS-CoV-2 the same is observed, once low antibody avidity is showed during early infection and it is detected strongly after 3 weeks of symptom onset in patients recovered from COVID-19^47^. Moreover, a study demonstrated that anti-spike avidity was directed correlated with higher neutralizing antibodies titers^45^.

Antibodies produced by patients that recovered from coronavirus disease have been used as a “molecular framework” to design antibody-based therapies^48^. Therefore, the antibody avidity and IgG subclass determination in COVID-19 has a good chance to highlight the clinical and immunological aspects of SARS-CoV-2 infection and further to determine protection after immunization. Thus, the study aimed to evaluate the kinetic, level, and avidity of antibodies IgG RBD-specific and the relationship of specific IgG subclasses to the severity of the disease.

## Results

### Demographic data, clinical aspects, and comorbidities

Forty-seven symptomatic patients tested positive for SARS-CoV-2 infection admitted at the Institute of Infectology Emilio Ribas (IIER; São Paulo, Brazil), between March and June 2020, enrolled in this study. The clinical characteristics of these patients are shown in Table 1. The median age was 59 years (IQR, 33–82 years); 61.7% were males, and 38.3%, females. The most common symptoms at illness onset were fever (39 [82.98%]), dry cough (39 [82.98%]), dyspnea (34 [72.34%]) and myalgia (20 [42.55%]). whereas obesity (25 [53.19%]), hypertension (23 [48.94%]) and diabetes (16 [30.04%]) were the most common comorbidities. Inpatients were seen at the Intensive Care Unit, and some of them, due to severity, required intubation (37 [78.72%]). The pharmacological treatment varied according to clinical features and coexisting disorders of each patient: 45 patients (95.74%) received antibiotics, 24 (51.06%) antiviral, 16 (30.04%) corticoids, 13 (27.67%) anticoagulants, and 9 (19.15%) chloroquine (Table 1). Most of the patients recovered from COVID-19 (35/47 [74.47%]), in which 25 (71.43%) developed the severe form of illness, while 10 (28.57%) developed the mild form of the disease. Obesity, hypertension, and diabetes were predominant comorbidities observed in both patients who recover or pass away***.***

**Table 1:** Characteristics of 47 enrolled patients.

Characteristics	All patients (N = 47)	Recovered (N = 35)	Dead (N = 12)
Age, median (range)	59 (33–82)	55 (33–77)	64 (53–82)
**Sex**			
Male	29/47 (61.7)	21/35 (60)	8/12 (66.67)
Female	18/47 (38.3)	14/35 (40)	4/12 (33.33)
**Clinical severity**			
Mild	10/47 (21.28)	10/35 (28.57)	0
Severe	37/47 (78.72)	25/35 (71.43)	12 (100)
**Symptoms**			
Fever	39/47 (82.98)	31/35 (88.57)	8/12 (66.67)
Dry cough	39/47 (82.98)	31/35 (88.57)	8/12 (66.67)
Dyspnea	34/47 (72.34)	25/35 (71.43)	9/12 (75)
Myalgia	20/47 (42.55)	16/35 (45.71)	4/12 (33.33)
Acute respiratory insufficiency	10/47 (21.28)	7/35 (20)	3/12 (25)
Headache	6/47 (12.77)	6/35 (17.14)	0
Thromboembolism	5/47 (10.64)	4/35 (11.43)	1/12 (8.33)
Diarrhea	4/47 (8.52)	3/35 (8.57)	1/12 (8.33)
Pneumonia	3/47 (6.39)	3/35 (8.57)	0
Chill	2/47 (4.26)	1/35 (2.86)	1/12 (8.33)
Asthenia	2/47 (4.26)	1/35 (2.86)	1/12 (8.33)
Odynophagia	1/47 (2.13)	1/35 (2.86)	0
Nasal obstruction	1/47 (2.13)	1/35 (2.86)	0
Respiratory distress	1/47 (2.13)	1/35 (2.86)	0
Dysuria	1/47 (2.13)	1/35 (2.86)	0
Sweating	1/47 (2.13)	1/35 (2.86)	0
Anosmia	1/47 (2.13)	1/35 (2.86)	0
Sore throat	1/47 (2.13)	1/35 (2.86)	0
Malaise	1/47 (2.13)	0	1/12 (8.33)
Hyporexia	1/47 (2.13)	0	1/12 (8.33)
**Coexisting disorders**			
Obesity	25/47 (53.19)	17/35 (48.57)	8/12 (66.67)
Hypertension	23/47 (48.94)	17/35 (48.57)	6/12 (50)
Diabetes	16/47 (30.04)	11/35 (31.43)	5/12 (41.67)
HIV	3/47 (6.39)	2/35 (5.71)	1/12 (8.33)
Deep vein thrombosis	3/47 (6.39)	2/35 (5.71)	1/12 (8.33)
COPD	3/47 (6.39)	2/35 (5.71)	1/12 (8.33)
Hypothyroidism	2/47 (4.26)	1/35 (2.86)	1/12 (8.33)
Prostatic hypertrophy	2/47 (4.26)	1/35 (2.86)	1/12 (8.33)
Stroke	2/47 (4.26)	1/35 (2.86)	1/12 (8.33)
Asthma	2/47 (4.26)	2/35 (5.71)	0
Pre-existing lesions	1/47 (2.13)	1/35 (2.86)	0
Tinea cruris	1/47 (2.13)	1/35 (2.86)	0
Sleep apnea	1/47 (2.13)	1/35 (2.86)	0
Epilepsy	1/47 (2.13)	1/35 (2.86)	0
Hepatitis B	1/47 (2.13)	1/35 (2.86)	0
Encephalitis	1/47 (2.13)	1/35 (2.86)	0
Cardiac insufficiency	1/47 (2.13)	1/35 (2.86)	0
Kidney transplant	1/47 (2.13)	1/35 (2.86)	0
None	4/47 (8.52)	4/35 (11.43)	0
**Treatment**			
Antibiotics	45/47 (95.74)	33/35 (94.29)	12 (100)
Antiviral	24/47 (51.06)	17/35 (48.57)	7/12 (58.33)
Corticoids	16/47 (30.04)	12/35 (34.29)	4/12 (33.33)
Anticoagulants	13/47 (27.67)	10/35 (28.57)	3/12 (25)
Chloroquine	9/47 (19.15)	7/35 (20)	2/12 (16.67)
Immunosuppressant	1/47 (2.13)	1/35 (2.86)	0
Vasodilator	1/47 (2.13)	1/35 (2.86)	0

### IgG RBD-specific antibodies detected by in house ELISA

**Figure 1:**
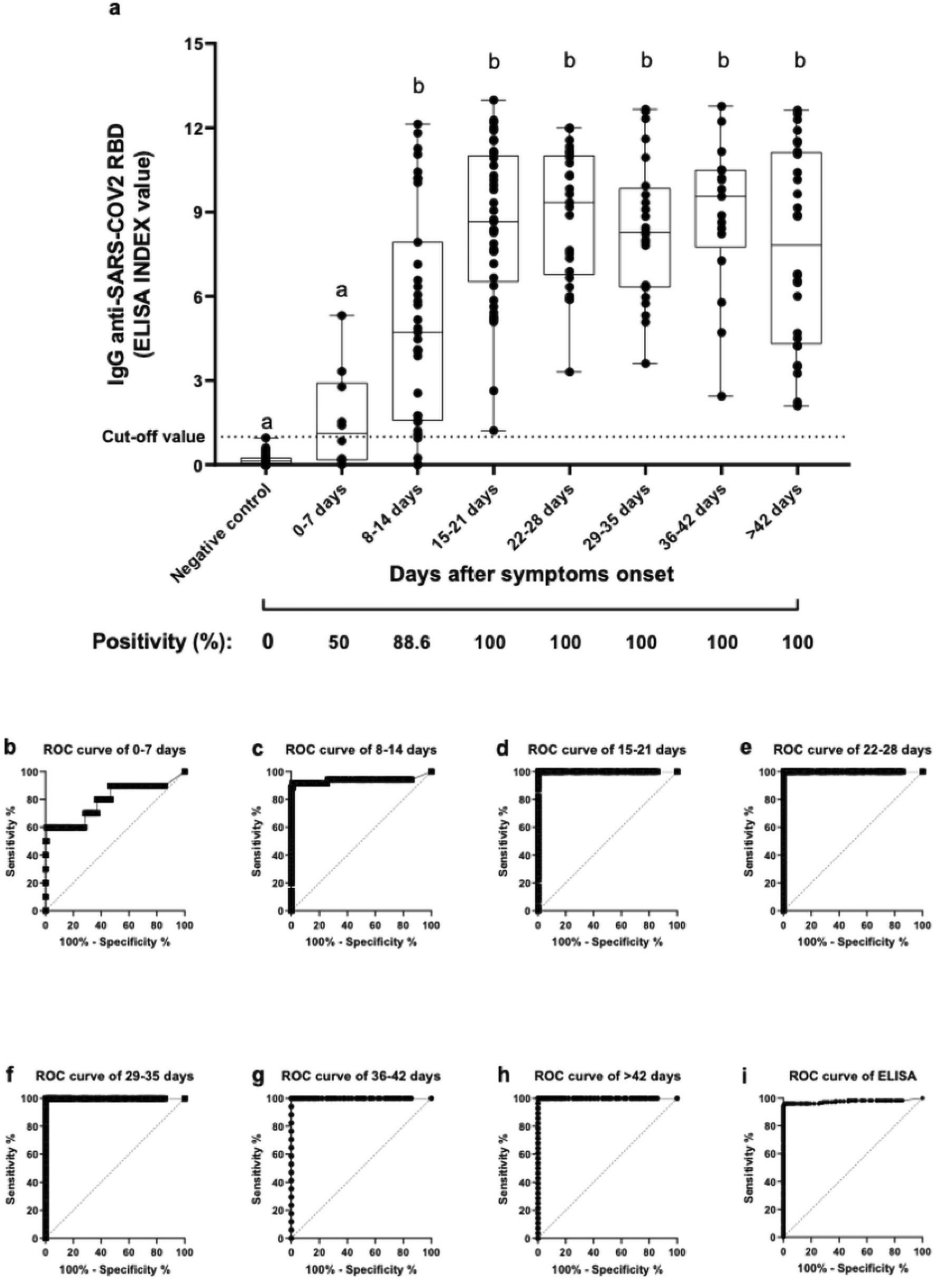
RBD-specific IgG antibody levels in symptomatic patients with SARS-CoV-2. (**a**). Levels of antibodies against SARS-CoV-2 RBD in patients at different times after symptom onset in 176 samples from 47 patients. Negative samples to COVID-19 (118 samples) were used as a negative control of ELISA. The ELISA data were expressed as ELISA Index (EI). Aligned dot plots and boxplots show EI values (dots), medians (middle line), third and first quartiles (boxes), while the whiskers display the minimum and maximum values. The dashed line indicates the cut-off value. Different letters (a, b) indicate statistical differences between each time in comparison with negative control determined by Kruskal–Wallis and Dunn’s multiple comparisons test (*P* < 0.05). The positivity in each time was expressed as a percentage (%). (**b**–**i**) Receiver operating characteristic (ROC) curves show the RBD IgG-ELISA's diagnostic performance in each time analyzed.

An adapted in house ELISA using RBD as coat antigen was performed to detect specific IgG in serum samples from SARS-CoV-2 symptomatic patients. A total of 294 serum samples were analyzed, being 176 serum samples collected on different days from 47 SARS-CoV-2 positive patients admitted to the Institute of Infectology Emilio Ribas (IIER) and 118 negative serum samples from Institute Adolfo Lutz (IAL) routine—(collected before 2019). Among SARS-CoV-2 positive patients, IgG RBD-specific antibodies were detected in 166 (94.32%) samples, while none SARS-CoV-2 negative controls (n = 118) showed reactivity (Fig. 1a). The sample from patients of different time points (intervals of 7 days) post-onset symptoms were analyzed (Fig. 1a) to assess the immunoreactivity throughout the time. Results had shown only 50% of samples (5 out of 10) from SARS-CoV-2 infected patients presented IgG RBD-specific positivity within the first-week post the onset of the symptoms, with no significant difference compared to negative controls.

Otherwise, from the second week after onset symptoms, the presence of IgG RBD-specific antibodies was observed among 88.6% of patient’s samples, reaching 100% of positivity in the next time-point, highlighting that the sensitivity of RBD-based ELISA improves according to the timing after the onset of clinical symptoms (Fig. 1b–i). The general immunoreactivity of RBD ELISA (including first and second week) resulted in 100% of specificity and 94.3% of sensibility in the population analysed (Fig. 1).

### IgG SARS-CoV-2 RBD-specific antibodies according to the outcome (recovered or died)

**Figure 2:**
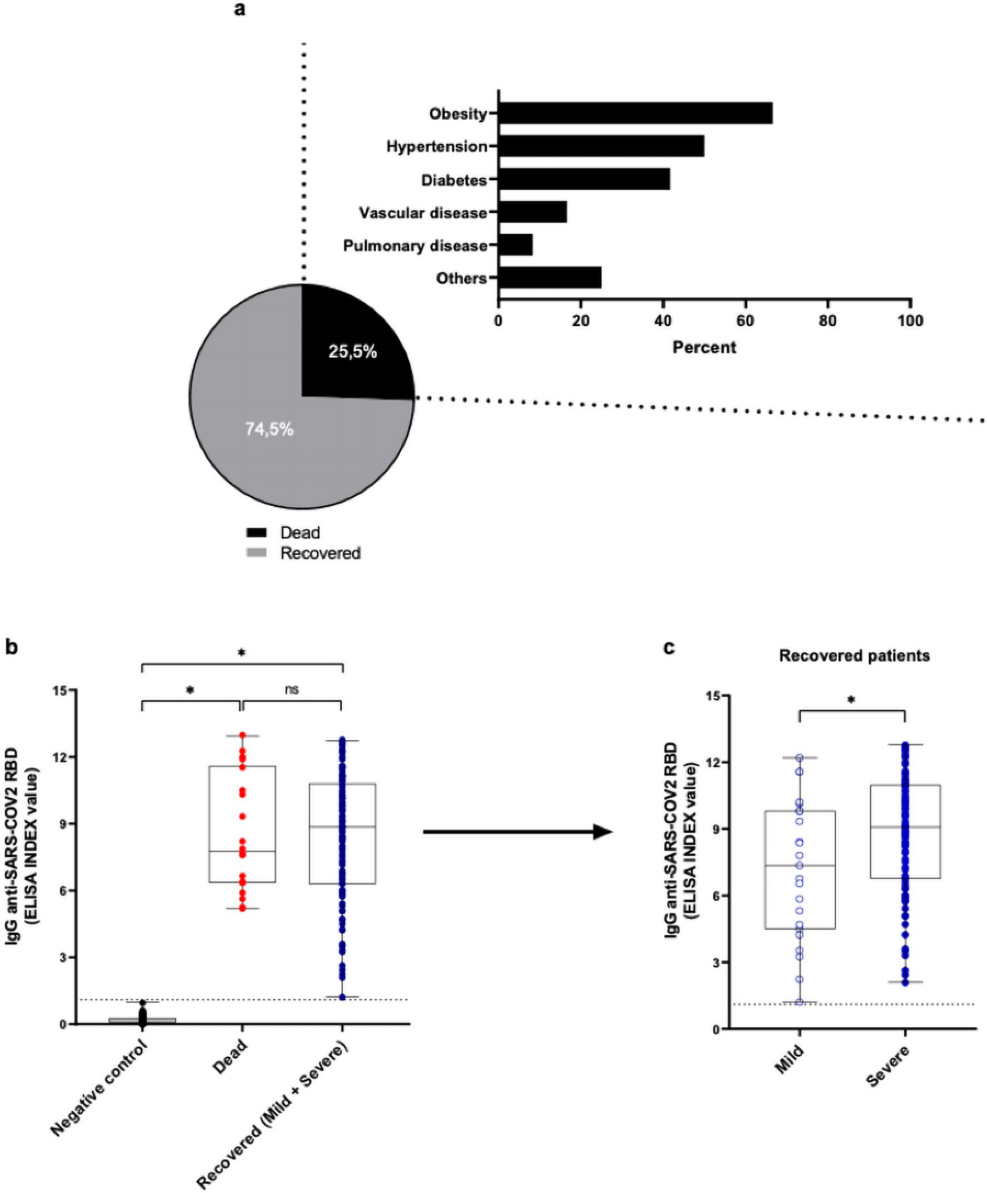
IgG antibody levels to SARS-CoV-2 RBD in patients who recovered (mild or severe) or who died with COVID-19. (**a**) Percentage of patients who recovered or died of COVID-19 and main comorbidities disease-associated. A detailed description of other comorbidities was shown in Table 1. (**b**) Levels of antibodies to SARS-CoV-2 RBD in patients who recovered (blue) or died (red) of COVID-19 or negative controls (black) were expressed as EI. (**c**) Paired analysis of RBD-specific IgG levels in survived patients displayed mild and severe clinical presentation of COVD-19. Aligned dot plots and boxplots show EI values (dots), medians (middle line), third and first quartiles (boxes), while the whiskers display the minimum and maximum values. The dashed lines indicate the cut-off values. Statistically significant differences were determined by Kruskal–Wallis and Dunn’s multiple comparisons test or Mann–Whitney test when appropriate (*P < 0.05). Not significant (ns).

We analyzed the IgG antibody response against SARS-CoV-2-RBD using samples from patients collected at least 15 days after symptoms onset, grouped according to the patient’s outcome: recovered (group 1, n = 35) and died (group 2, n = 12). The significant comorbidities observed in patients who died were obesity (8/12 [66.67%]), followed by hypertension (6/12 [50%]) and diabetes (5/12 [41.67%]) (Fig. 2a). The group of patients who died displayed a slightly lower anti-RBD IgG level than the recovered one, although no statistical differences were observed in both groups (Fig. 2b). Interestingly, recovered patients with severe COVID-19 had higher anti-RBD IgG levels than patients with mild disease (P < 0.05, Fig. 2c).

### IgG antibody avidity to SARS-CoV-2 RBD

**Figure 3:**
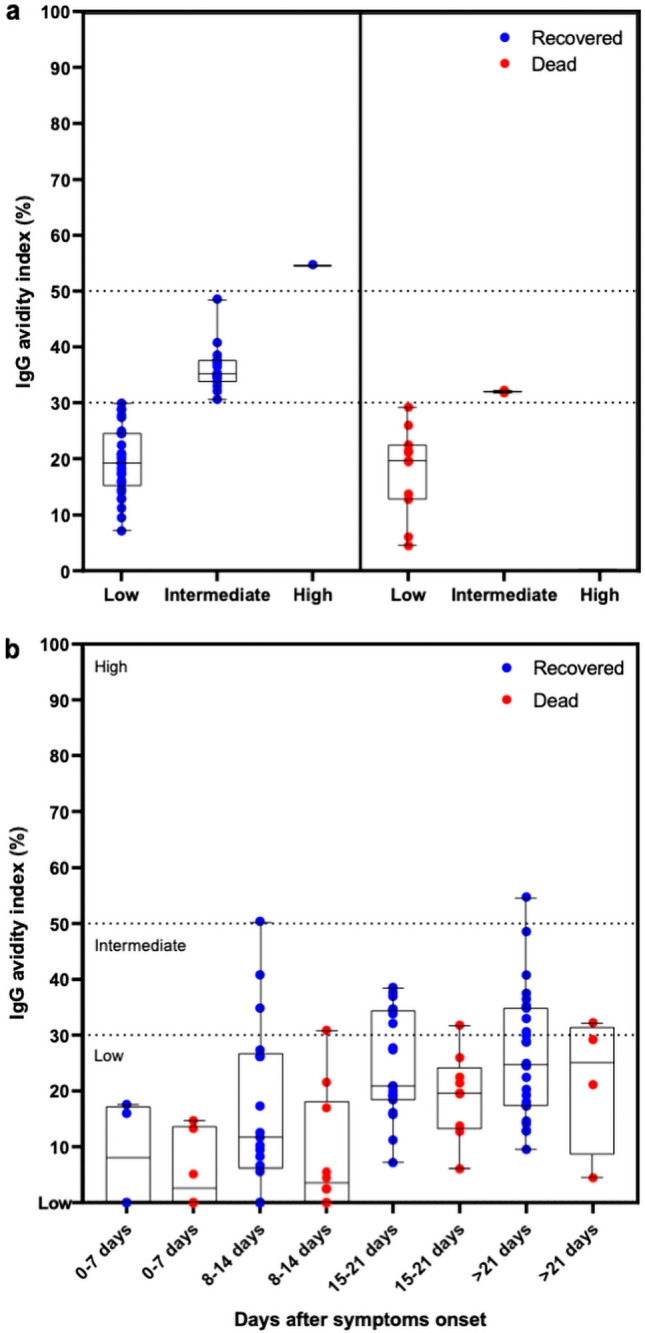
Binding strength of IgG antibody to SARS-CoV-2 RBD (avidity) in COVID-19 patients. (**a**) IgG antibody avidity in patients who recovered (blue) or who died (red) with COVID-19. The IgG antibody avidity was expressed as the Avidity Index (AI %). The dash lines indicate the avidity range (low, intermediate, and high). (**b**) IgG antibody avidity to RBD concerning the time after symptom onset in recovered or dead groups. Aligned dot plots and boxplots show AI values (dots), medians (middle line), third and first quartiles (boxes), while the whiskers display the minimum and maximum values. Statistically significant differences were analyzed by Kruskal–Wallis and Dunn’s multiple comparisons test or Mann–Whitney test when appropriate (*P* < 0.05).

Samples collected from 15 days after onset of symptoms from patients with COVID-19 were evaluated for binding strength of IgG antibodies to RBD protein. The majority of patients analyzed displayed low antibody avidity, with 64.4% of the samples of patients who recovered from the disease and 84.6% of those who died in this avidity range (Fig. 3a). Intermediate antibody avidity was observed in 33.3% and 15.4% in the recovered or died group, respectively. Remarkably, one sample from a patient that recovered displays high avidity to SARS-CoV2 RBD, despite a relatively short period (weeks) posts the onset of the symptoms. When the samples were analyzed in parallel, in each avidity range (low, intermediate, high), no differences were detected between patients who recovered or died from COVID-19 (Fig. 3a). Similarly, when samples from patients who recovered or died were analyzed each week (7-day intervals) comparatively, no statistically significant difference was observed (Fig. 3b).

### Antibody subclasses response in symptomatic patients with COVID-19

**Figure 4:**
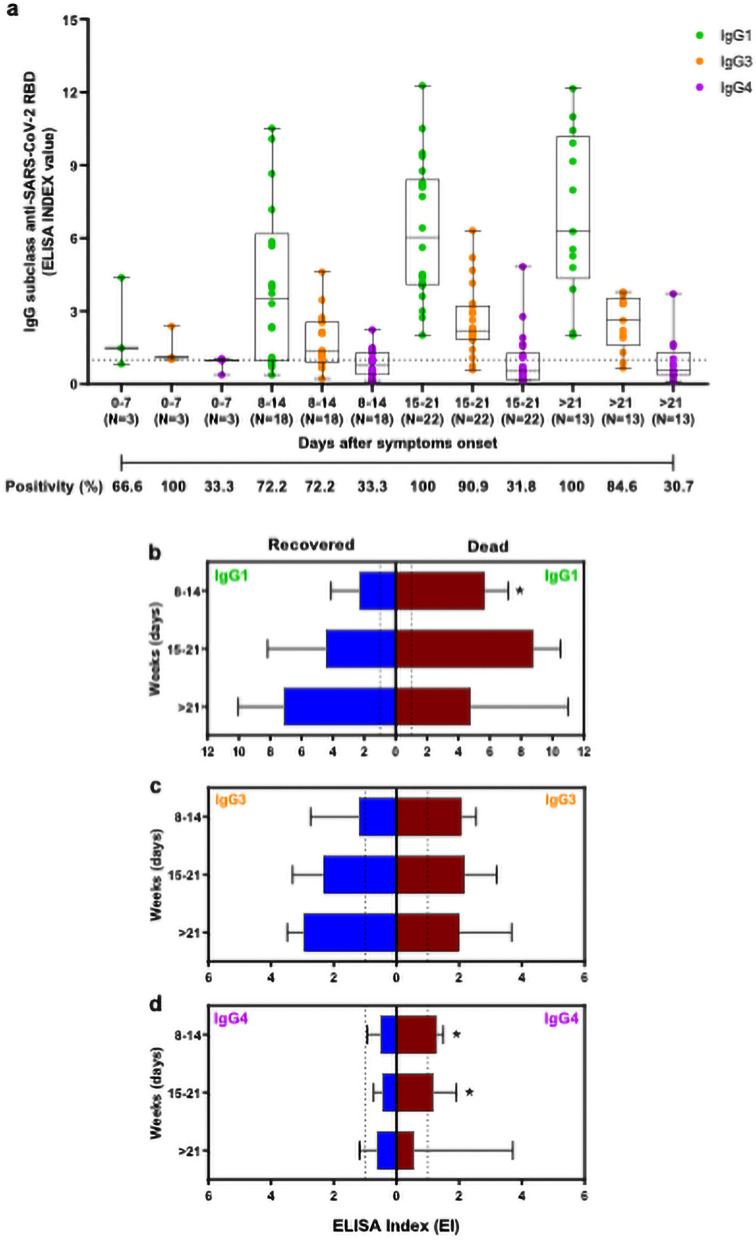
Analyses of IgG subclasses to SARS-CoV-2 RBD in patients with COVID-19. (**a**) Kinetics of IgG1, IgG3, and IgG4 in COVID-19 patients in different time-points post the onset of the symptoms. The data were expressed as EI (dots), medians (middle line), third and first quartiles (boxes), while the whiskers display the minimum and maximum values. Numbers of patients (N) are indicated underneath. The positivity, in each time and respective IgG subclasses, was indicated as a percentage (%). Comparison of IgG1 (**b**), IgG3 (**c**), and IgG4 (**d**) in patients who recovered (blue) and who died (red) with COVID-19. Boxplots show the median of EI values and interquartile ranges. The dashed lines indicate the cut-off values. Statistically significant differences between groups were determined by the Mann–Whitney test each time (*P < 0.05).

IgG subclasses maybe become relevant in clinical conditions of COVID-19, considering that IgG subclasses to SARS-CoV-2 are a key to a better clinical condition, with IgG1 and IgG3 being more abundant in patients that are in the mild case and do not die. Our data reveal an increase of IgG1 and IgG3 levels since the 8th day after symptoms onset (Fig. 4a), while IgG4 levels maintained less detectable during the study period. Anti-RBD IgG1 positivity oscillated from 66.6 to 100%, reaching the highest values in the third and fourth week of analysis. The positivity of anti-RBD IgG3 ranged from 66.6 to 90.9%, whereas IgG4 presented positivity from 66.6 to 46.1%, with higher values for IgG3 and IgG4 positivity observed in the third and fourth weeks, respectively.

When patients who died and who recovered were analyzed in parallel, it was possible to identify a higher anti-RBD IgG1 response in patients who died compared to those who recovered during 8–14 days after symptoms onset (*P* < 0.05), as illustrated in the Fig. 4b. No statistical differences were observed in IgG3 levels from recovered and dead patients (Fig. 4c). Surprisingly, patients who died during 8–14 and 15–21 days also showed higher anti-RBD IgG4 levels in comparison with the recovered (P < 0.05) (Fig. 4d), suggesting that some life-threatening patients can elicit IgG4 to RBD response in the first weeks of symptoms onset.

## Discussion

SARS-CoV-2 exhibits a high infectivity capacity; the new disease reached million cases worldwide, being observed more than 4  million deaths^7^. Brazil is entering through sucessive  waves of COVID-19, and some restrictions are being imposed. However, the number of cases is still  worrying. In the search for vaccines and a useful serological assay to trial even asymptomatic people, SARS-CoV-2 infection remains a significant problem affecting Brazil. Although qPCR to detect the viral genome is highly sensitive diagnostic assay in the initial phase of COVID-19, additional screening methods that detect the presence of SARS-CoV-2 infection, including serological tests, can be highly advantageous to ensure timely diagnosis^21,24,31,40,41,49,50^ in a pandemic. Several studies have reported the profile of the antibody response to SARS-CoV-2, which includes the broad clinical spectrum of COVID-19. Many aspects of the humoral immune response in COVID-19 remain obscure^26,51–53^, particularly concerning clinical utilities of serological testing in symptomatic and hospitalized patients.

In this attempt, we evaluated the IgG response in hospitalized patients with mild and severe clinical manifestation, showing that RBD-specific IgG responses begins 4 days after symptoms onset, reaching the plateau at 15 days. The average time to detect IgG against SARS-CoV-2 based on RBD IgG-ELISA was similar to what was found in other studies, regardless of the serological assay and SARS-CoV-2 antigen used^26,51–53^. In our in-house ELISA, high immunoreactivity was observed with 100% specificity and 94.3% of sensibility, suggesting that RBD IgG-ELISA could be used to assess SARS-CoV-2 infection in patients 15 day-post onsets of symptoms. Wolf et al. observed that hospitalized COVID-19 patients showed higher leves of antibodies when compared to outpatients cases, suggesting an association between illness severity and antibody production^48^.

Interestingly, in our study, survivors who developed the severe form of illness displayed higher anti-RBD IgG levels compared to patients with mild disease, also lighting that clinical presentation of disease may induce substantial differences in IgG response. For this purpose, we also investigated the anti-RBD IgG levels in recovered patients compared to those who died. Although no statistical differences were observed, a slightly lower anti-RBD IgG level was observed in patients who died compared to the recovered ones. The divergence observed in our study may be due to differences in the number of serum samples analyzed and the time of blood collection, since a limited number of samples was obtained from critically ill patients who quickly progressed to death by COVID-19.

Between the comorbidities observed in patients who died, obesity was more frequently observed in our study. The high percentage of obese patients who died with COVID-19 is in concordance with previous studies^54,55^. Obesity is a factor that directly associated chronic activation of innate immune system cells and consequent local and systemic inflammation^56^. B cells from obese patients express leptin induced-activation markers (TNF-α, TLR4, micro-RNAs) that correlate reduced B-cell functions^49,51,52^. Therefore, obesity and COVID-19 share common elements of the inflammatory process (and possibly also metabolic disturbances), exacerbating SARS-CoV-2 infection in the obese^53^, leading these individuals to severe COVID-19, even to death.

In the present study, high levels of anti-RBD IgG were detected in both groups of patients who died or survived. However, these findings are not enough to support the hypothesis that these individuals displayed either an extended-lasting protective humoral response against SARS-CoV-2 or neutralizing antibodies in convalescent plasma. Our study has some limitations in this context, since it was not possible to assess the presence and neutralizing antibodies. Interestingly, a possible association between SARS-CoV-2 spike antibody avidity with neutralizing IgG titer, as a potential screening parameter for identifying optimal convalescent plasma donors, was previously proposed^45,57^. Likewise, high-avidity antibodies toward another virus capable of blocking receptor binding were protective and promoted virus neutralization^58–68^, indicating that antibody avidity maturation could be, at least in part, associated with the production of protective neutralizing antibodies to SARS-CoV-2. In addition, the efficient rupture of the strong interaction stablished between SARS-CoV-2 RBD and human ACE2 protein has been proposed to require the high-binding affinity antibodies toward multiples sites of RBD (at least two molecular epitopes) which highlights the importance of functional affinity in blockage SARS-CoV-2 infection^14^. Interestingly, other viral infections such as, cytomegalovirus^63,69^, Dengue^70^ and Vesicular Stomatitis Virus^71^ require antibodies with high avidity, instead of high titers, to confer a protective effect. Similar result was also reported in study evaluating immune response to malaria’s vaccine^72^. These findings encourage the use of avidity determination for SARS-CoV-2 infections.

Although our study has limited data on temporal dynamics (< 45 days) to correlates SARS-CoV-2 antibody avidity with the illness severity, it was observed that the majority of patients who had symptoms showed low avidity. Our data is in agreement with what was reported to SARS-CoV-2 infection, ﻿with low IgG antibody avidity during the 50 days after symptoms onset^45,73^, however, it was not observed in other studies which considered anti-nucleocapsid and anti-spike^45,55^.

Similarly, low antibody avidity was also observed ﻿in early infection and augmented within the first month of symptom onset in SARS outbreaks^49^. It is noteworthy that one-third of the patients who recovered had intermediate avidity of IgG antibodies to RBD, despite a relatively short period post the onset of the symptoms. On the other hand, approximately one-sixth of the patients who died produced intermediate avidity, suggesting that IgG avidity may be useful for monitoring hospitalized patients with COVID-19 in association with other serological markers. As was expected, IgG antibody avidity was low during initial infection and increased with time, although no statistical differences were observed between patients who died and recovered in the time-points post the onset of the symptoms.

The IgG1 and IgG3 subclasses represent the predominant antibody responses to several viral diseases^44,74^, and recently it was also associated with the new SARS-CoV-2 infection^43,50^. IgG1 and IgG3 responses are related to immune functions such as viral neutralization, opsonization, and complement activation in viral respiratory infection^44^. We analyzed IgG isotypes ﻿to SARS-CoV-2 RBD in sera from patients with COVID-19. As it was expected, a robust antibody response of IgG1 and IgG3 specific to RBD occurred predominantly in comparison with minor IgG4 responses. Likewise, Suthar et al. demonstrated that COVID-19 patients analyzed in USA produced RBD-specific IgG1 and IgG3 early during acute infection, with no detectable IgG2 or IgG4^43^. Similar antibody responses were also reported by Mazzini et al. in Italy, with a strong reactivity for IgG1 and IgG3 in sera from positive patients for SARS-CoV-2 infection^50^.

The comparative analysis of IgG subclasses in serum samples from COVID-19 patients who died revealed a higher level of RBD-specific IgG1 when compared to those who survived during 0–8 days after symptoms onset. However, this difference was not maintained in more advanced times of the onset of the symptoms. Although the production of RBD-specific IgG1 is consistent with activation of Th1 lymphocytes^75^, this difference in the first week cannot be explained simply by evaluating different subpopulations of T helper cells, but may involve other factors, including sample bias, differences in individual immune responses, and/or early viral load. Also, no statistical differences were observed in IgG3 levels between patients who died and recovered. Surprisingly, we also noticed higher levels of RBD-specific IgG4 in sera from patients who died when compared to survivors in the second and third weeks. In our analysis, more that half serum samples of patients who progressed to death showed positivity to RBD-specific IgG4 antibodies, whereas most patients who recovered from COVID-19 were IgG4 negative to SARS-CoV-2 RBD in the same window of time. The IgG4 biosynthesis is known to be induced under conditions of increased IL-10 cytokine^76^ having as a primary source several immune cells, including Th2 cells, regulatory T cells (Treg), or even regulatory B cells (Breg). Patients with severe COVID-19 display sustained inflammation and continued production of various anti- and pro-inflammatory cytokines (cytokine storm syndrome)^37^, including the IL-10 production that may be associated with induction of IgG4 antibodies in severe COVID-19. Although substantial knowledge about the antibody response has already been generated nowadays for COVID-19, further studies are necessary to understand the role of IgG4 antibodies in COVID-19 pathophysiology.

In conclusion, the present study constitutes the effort to clarify the kinetics of IgG antibodies, avidity, and subclasses against SARS-CoV-2 RBD in symptomatic patients with COVID-19 in Brazil, highlighting the importance of IgG antibody avidity in association with IgG4 detection as a laboratory tool in the follow-up of hospitalized patients with more significant potential for life-threatening conditions in the population analyzed.

## Methods

### Study design

**Figure 5:**
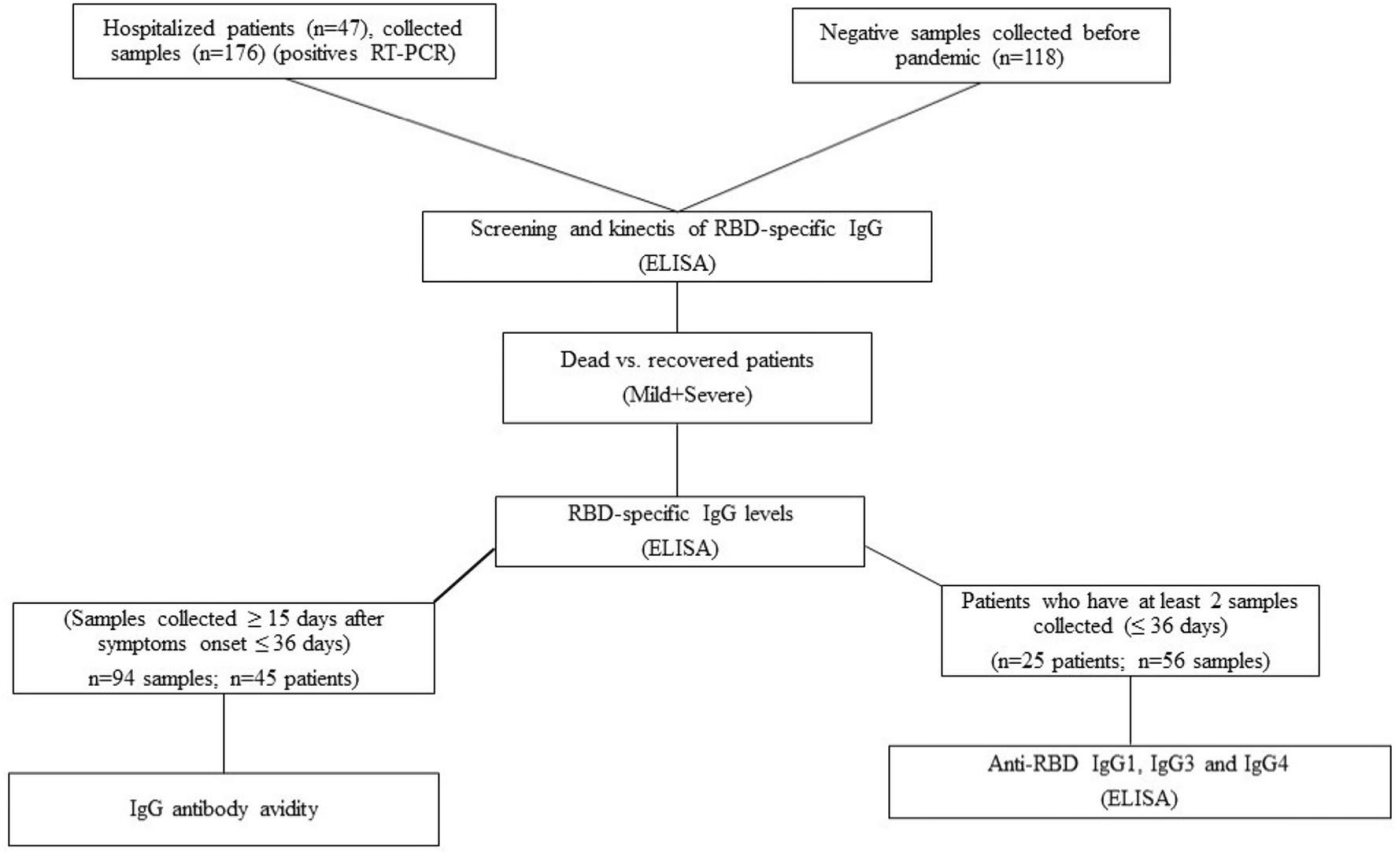
Flow chart representative of the proposed study design.

Forty-seven symptomatic patients (there was no participation of subjects under 18 years old) tested positive for SARS-CoV-2 infection by RT-PCR were admitted at Institute of Infectology Emilio Ribas (IIER) São Paulo, Brazil, between March and June 2020 were enrolled in this study. All patients who presented typical symptoms of illness such as fever, dry cough, dyspnea, myalgia, etc., were classified as mild or severe, according to IIER protocols previously established for COVID-19. Blood samples were taken at different time points until either the patient was discharged or died. A total of 294 serum samples were used from patients (136 collected from patients who were discharged and 40 from patients who died) and 118 serum samples SARS-CoV-2 negative, collected before September 2019 and selected from Institute Adolfo Lutz (IAL) routine, were analyzed (Fig. 5). SARS-CoV-2 negative serum samples have a documented history of other viral infections (HIV-1, HIV-2, Hepatitis B, Hepatitis C, Dengue, Chikungunya, Yellow fever) non-related with any coronavirus and bacterial infections (*Treponema pallidum*, *Mycoplasmas pneumoniae*).

### Ethical approvements

The results described in this manuscript is part of a project approved by research ethical committee from Institute of Infectology Emilio Ribas, CAAE: 32264120.5.2001.0061. The free and informed consent form was obtained from the research subjects and when it was impossible to obtain the signature, the confidentiality term was used for the remaining samples sent to the laboratory for routine examinations. There was no participation of subjects under 18 years old. Demographic data, Clinical and hematological conditions at first attendance were obtained for all patient included in the study. All methods were performed in accordance with the relevant guidelines and regulations.

### SARS-CoV-2 RBD recombinant antigen

The receptor-binding domain (RBD) antigen, derived from SARS-CoV2 Spike protein, was kindly donated by Dr. Florian Krammer^24,25^, from Icahn School of Medicine, Mount Sinai, Nova York, NY, EUA.

### Indirect ELISA for detection of SARS-CoV-2 RBD-specific IgG antibodies (ELISA-RBD)

IgG RBD-specific antibodies were detected by Enzyme-Linked Immunosorbent Assay (ELISA) using an adapted protocol previously described by Stadlbauer et al^25^*.* Briefly, high-binding 96-well plates (Nunc MaxiSorp™ flat-bottom) were coated with 50 µl per well of 2.5 µg per ml of RBD protein diluted in PBS at 4 °C overnight. The next morning, plates were washed four times with PBS supplement with 0.01% Tween 20 (PBST). All wash steps were performed using an ELISA plate washer (Washwell plate, Robonik, Thane, India). After, 200 µl per well of 5% skim milk powder diluted in PBST was added to the plates and incubated for 2 h at room temperature as a blocking solution. After blocking, plates were washed four times with PBST and incubated with 200 µl per well of each serum sample, in duplicate, diluted 1:200 in PBST containing 1% of skim milk for 1 h at room temperature. Next, plates were washed four times with PBST and 50 µl of a 1:15,000 dilution of goat anti-human IgG (whole molecule)—Horseradish Peroxidase (HRP) antibody (Sigma-Aldrich) diluted in PBST containing 1% skim milk was added to wells, and the plates incubated for 1 h at room temperature. Plates were rewashed with PBST and incubated for 10 min with 100 µl of One Step-TMB (3,3′,5,5′-tetramethylbenzidine) (Scienco, Santa Catarina, Brazil). The reaction was stopped by the addition of 50 µl per well of 1 N sulfuric acids. The optical density at 450 nm (OD450) was measured using a Multiskan MS plate reader (Labsystems). The cut-off value was established based on the maximum sensitivity and specificity using a two-graph receiver operating characteristic (TG-ROC) analysis as previously described^77^. Antibody titers were expressed as ELISA index (EI), according to the following formula: EI = OD sample/cut-off. Samples with EI values > 1.0 were considered positive.

### Evaluation of IgG antibody avidity to SARS-CoV-2 RBD

Serum samples were submitted to IgG avidity ELISA using potassium thiocyanate (KSCN) as a chaotropic chemical reagent as previously described^78^ to assess the interaction between them IgG antibodies and RBD. Following the above described ELISA-RBD, an extra step was performed after incubation with serum. Briefly, after serum incubation, plates were washed four times with PBST, and wells were treated in the presence or absence of KSCN 1.5 M (200 µl/well) for 20 min at room temperature. After, plates were washed four times and incubated with goat anti-human IgG HRP-antibody (1:15,000) for 1 h at room temperature. The reaction was revealed with One Step-TMB solution as described to indirect ELISA. Avidity Index (AI%) was expressed as follows: AI% = (OD mean value from KSCN treated sample divided by the OD mean value from the non-treated) multiplied by 100. AI values above 50% were considered high antibody avidity; between 31 to 49%, intermediate avidity, and below 30%, low avidity^79^.

### Measurement of IgG subclasses specific to SARS-CoV-2 RBD

ELISA was used to detect IgG1, IgG3, IgG4 using a protocol previously described^80^. Briefly, 96-well plates (Nunc MaxiSorp™ flat-bottom) were coated with 50 µl per well of 2.5 µg per ml of RBD protein diluted in PBS at 4 °C overnight. Plates were then washed four times with PBS with 0.01% Tween 20 (PBST) using a Washwell Plate (Robonik). Samples were blocked with 100 µl per well of 1% Bovine Serum Albumin (BSA) diluted in PBST for 1h at 37 °C. After blocking, plates were washed four times with PBST and incubated with serum diluted 1:50 in PBST-0.1% BSA, in duplicate, for IgG1 detection. For IgG3 and IgG4 subclasses, serum was diluted 1:5 in the same solution, and the assay was also performed in duplicate. Thus, samples were incubated for 2 h at 37 °C, washed four times, and incubated with the respective biotinylated secondary antibodies (Sigma): goat anti-human IgG1 (1:1000), anti-human IgG3 (1:1000), or anti-human IgG4 (1:1000) diluted in PBST-0.1% BSA for 1 h at 37 °C. Plates were rewashed four times and incubated with 50 µl of 1:500 streptavidin–peroxidase (Sigma/Merck) diluted in PBST-0.1% BSA (Sigma) for 30 min at 37 °C. After the final washing step (four times), samples were revealed with ABTS (Sigma/Merck). The optical density at 405 nm (OD405) was measured using a Multiskan MS plate reader (Labsystems). Cut-off of reaction was calculated using optical density values of negative pools plus three standard deviations as described^80^. Antibody titers were expressed as ELISA index (EI), and values > 1.0 were considered positive.

### Statistical analyses

The data were evaluated for normal distribution by D’Agostino and Pearson, Shapiro–Wilk, Kolmogorov–Smirnov normality tests. Statistically significant differences among antibody IgG levels, antibody avidity, and IgG subclasses to SARS-CoV-2 RBD were determined by Kruskal–Wallis and Dunn’s multiple comparisons test or Mann–Whitney test when appropriate. *P* values < 0.05 were considered statistically significant^81^. Statistical analyses and graphics were performed using the GraphPad Prism v. 8.0 (GraphPad Software, San Diego, USA).
